# Sub-clinical rheumatic heart disease (RHD) detected by hand-held echocardiogram in children participating in a school-based RHD prevention program in Tanzania

**DOI:** 10.1186/s12872-023-03186-y

**Published:** 2023-03-25

**Authors:** Pilly Chillo, Reuben Mutagaywa, Deogratias Nkya, Marina Njelekela, Gideon Kwesigabo, Febronia Kahabuka, Vanessa Kerry, Appolinary Kamuhabwa

**Affiliations:** 1 Department of Internal Medicine, Section of Cardiology, School of Medicine, Muhimbili University of Health and Allied Sciences, Dar es Salaam, Tanzania; 2 Department of Paediatric and Child Health, Muhimbili University of Health and Allied Sciences, Dar es Salaam, Tanzania; 3 Department of Physiology, Muhimbili University of Health and Allied Sciences and Deloitte Consulting Limited, Dar es Salaam, Tanzania; 4 Department of Epidemiology and Community Health, Muhimbili University of Health and Allied Sciences, Dar es Salaam, Tanzania; 5 Department of Orthodontics Paedodontics & Community Dentistry, Muhimbili University of Health and Allied Sciences, Dar es Salaam, Tanzania; 6 Department of Global Health and Social Medicine, Seed Global Health Partnerships, Boston, USA; 7 Harvard Medical School, Center for Global Health, Mass General Hospital, Boston, MA USA; 8 Department of Clinical Pharmacy and Pharmacology, Muhimbili University of Health and Allied Sciences, Dar es Salaam, Tanzania

**Keywords:** Rheumatic heart disease, Sub-clinical rheumatic heart disease, Sub-sahara Africa, Tanzania, Rheumatic fever

## Abstract

**Background:**

Rheumatic Heart Disease (RHD) continues to cause suffering and premature deaths in many sub-Saharan Africa (SSA) countries, where the disease is still endemic. RHD is largely preventable and determining its community burden is an important critical step in any RHD prevention program.

**Methods:**

We conducted a cross-sectional study of 5–16 years old pupils from 11 primary schools participating in an RHD prevention program in 4 districts in Tanzania, between 2018 and 2019. At the school, all children were invited to participate after receiving consent from their parents/guardians. Participating children filled a questionnaire and were auscultated for cardiac murmurs. Echocardiographic screening was done by two experienced cardiologists, using a hand-held machine (V-Scan, GE®). All positive screening tests were stored for further examination by the same two cardiologists to reach to a consensus of definite, borderline or no RHD, using a modified World Heart Federation (WHF) criterion.

**Results:**

Of the 6895 children invited, 4738 (68.7%) were screened and 4436 (64.3%) had complete data. The mean (SD) age was 10.04 (2.43) years, and 2422 (54.6%) were girls. Fifty three (1.2%) children were found to have a murmur. The proportion of children with trace or mild valvular regurgitation, sub-valvular/chordal thickening and valvular thickening/deformity were 8.3%, 1.3%, and 1.0%, respectively. Sub-clinical RHD was found in 95 children (59 definite and 36 borderline), giving a prevalence of 2.1%, [95% CI 1.7% – 2.6%]. Sub-clinical RHD was independently associated with female sex (aOR 1.83, 95% CI 1.18–2.85, p = 0.007), older age groups (aOR 1.73, 95% CI 1.10–2.72, p = 0.018 for age group 11–14 years; and aOR 3.02 95% CI 1.01–9.05, p = 0.048 for age group 15–16 years), as well as presence of a cardiac murmur, aOR 5.63 95% CI 2.31–13.69, p < 0.0001. None of the studied socio- or economic factors was associated with the presence of sub-clinical RHD in this study.

**Conclusion:**

The prevalence of sub-clinical RHD among primary school children in Tanzania is 2.1%, similar to previous reports in SSA. Efforts to prevent and control RHD in our communities are highly warranted.

## Introduction

Rheumatic heart disease (RHD) is the most common acquired heart disease in children and young adults. According to the most recent Global Burden of Disease estimates, RHD affects an estimated 40.5 million people [1], and globally up to 80 million people may have asymptomatic RHD [2]. The disease accounts for approximately 275,000 deaths annually, and although almost eradicated in developed countries, RHD continues to affect many children and young adults in SSA countries, including Tanzania [3, 4]. RHD can largely be prevented when appropriate control programs are implemented focusing on community awareness, appropriate diagnosis and treatment of its precursors, namely streptococcus sore throat, acute rheumatic fever (ARF) and sub-clinical RHD [5].

Gathering data on the community burden of RHD is an important critical step before any RHD prevention program is initiated [6]. Globally, there is currently an increased interest to control or where possible eradicate RHD, resulting from an intensified advocacy in regions where RHD is still endemic [7, 8]. In SSA, several recent screening surveys on community burden of RHD have been published [9–17], and the findings show that subclinical RHD occurs in 1–3% of 5–17 years old children in the region [9–18]. Of these, only a few studies have reported the screening to be part of an ongoing RHD prevention program [11, 13].

In its natural history form, sub-clinical RHD occurs 5–15 years before the clinical manifestations of RHD ensues [19]. The long latent phase of RHD offers an opportunity for prevention before the disease becomes clinically overt. The World Health Organization (WHO) recommends screening as an effective way to detect the disease in early stage when secondary prophylaxis can be offered in those who would benefit the most [20]. Indeed, a recent clinical trial from Uganda confirmed that secondary prophylaxis given to children with sub-clinical RHD prevents disease progression [21]. Screening is also a way of raising awareness to policy makers who need to be on board in any RHD prevention program.

Traditionally, auscultation was used to screen for subclinical RHD [22, 23]. This method has however been superseded by screening using a portable echocardiography, which has shown to be up to 10 times more sensitive than auscultation [11, 13]. Therefore, portable echocardiography has been the mainstay of subclinical RHD screening [24]. However, increasing evidence suggests that the more compact hand-held echocardiogram may be as sensitive as the conventional portable echocardiogram, especially when used by experts [25–28]. The relatively low cost of the hand-held echocardiogram has an added advantage by permitting widespread RHD screening, especially in low resource settings where the use of the more expensive portable echocardiogram is a limiting factor [29].

As part of the research agenda for the establishment of the East African Centre of Excellence for Cardiovascular Sciences (EACoECVS) within the Muhimbili University of Health and Allied Sciences [30], RHD was identified as a priority disease due to its persistent high morbidity and mortality in Tanzania [23, 31, 32]. Beginning in 2018 the EACoECVS has been implementing a community based RHD prevention program which has several components including: obtaining community prevalence and risk factors of RHD, developing RHD health education and awareness materials for Tanzanian primary health care workers, pupils and teachers as well as delivering health education on prevention of RHD in these population groups. The project involves 11 schools in 4 districts (Bagamoyo, Kisarawe, Babati and Kiteto) in Tanzania representing semi-urban and rural populations. Here we present findings of an RHD screening survey we conducted between 2018 and 2019 that aimed at determining the community burden and associated risk factors of RHD in these communities.

## Materials and methods

### Study design, population and area

**Fig. 1:**
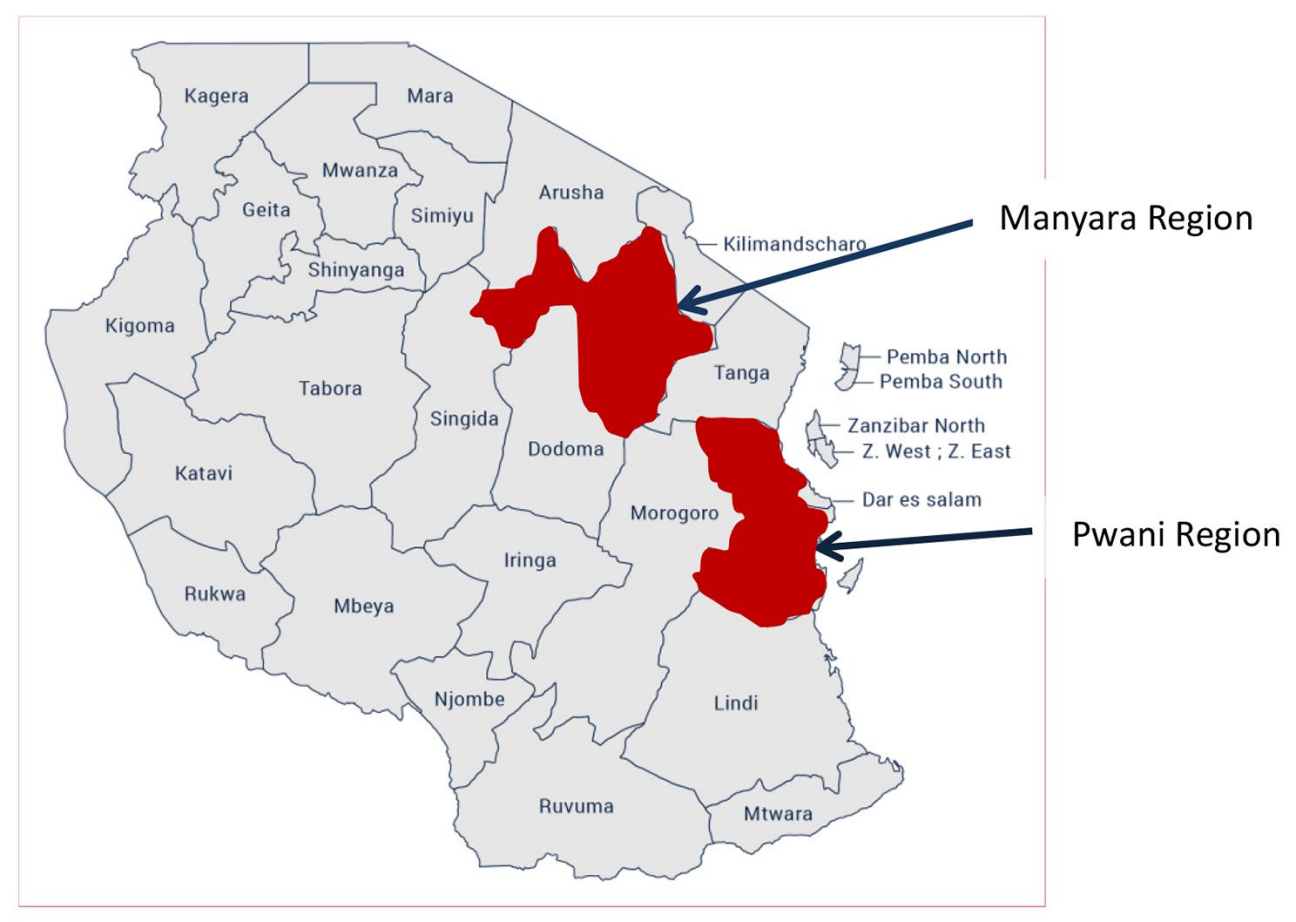
Map of Tanzania showing study area

This was a cross-sectional school-based survey conducted among public primary school children between the ages 5–16 years. The survey was conducted in Mainland Tanzania. In the 2012 census, Mainland Tanzania had a total population of 46.3 million, of which young population 0–17 years was 21.8 million (47.1%). The country has 25 administrative regions which are grouped into seven geographical zones. This study was conducted in two regions Pwani (Eastern zone) and Manyara (Northern zone), Fig. 1.

### Sample size calculation

Sample size calculation was based on reported prevalence of RHD using echocardiography screening in school children in SSA of 1–3%. The sample size was calculated to be 4,000 school children between the ages 5–16 years with type 1 error of 0.05 and a power of 80% for an expected diagnosis rate of 2 cases per 100 school children screened.

### Sampling procedure

A multi-stage sampling procedure was used. Two zones were randomly selected from the list of the seven geographical zones in Mainland Tanzania, and a further simple random selection was applied to obtain one region from each zone. At the region, all districts were listed in alphabetical order and then two districts were randomly chosen from each region.

At the district level, a list of all public primary schools was obtained from the District Administrative Office. Schools were then stratified as semi-urban or rural depending on their locations, and from each district, primary schools representing semi-urban and rural areas (in 1:2 ratio) were randomly selected to obtain schools included in the program. In one of the districts (Kiteto), only 1 rural school was surveyed due to logistical problems. At the school, all children aged 5–16 years were invited to participate through letters distributed to their parents/guardians. Only children whose parents/guardians gave a written consent were allowed to participate in the survey. Furthermore, children had to give a verbal assent before they were enrolled in the survey. In total, 11 public primary schools were included in the survey as summarized in Table 1.

**Table 1:** List and distribution of Primary Schools involved in the study

Region	District	Selected schools	
		Semi-urban	Rural
Manyara	Kiteto	Kaloleni	Osteti
	Babati	Magugu	Mbugwe, Dareda Kati
Pwani	Bagamoyo	Kiromo	Buma, Kondo
	Kisarawe	Mloganzila	Masaki, Masanganya

### Data collection

#### Socio-demographic and economic data

A structured questionnaire was used to acquire data on socio-demographic background and selected socio-economic indicators. Socio-demographic indices of age, sex, parents’ or head of household education and occupation were asked. Furthermore, details of household characteristics including roofing, floor, and wall materials were asked. In the medical history of the questionnaire, children were also asked if they have been on any regular monthly injections (as a proxy for secondary prophylaxis of RHD using Benzathine Penicillin). All children were asymptomatic at screening.

#### Screening for subclinical rheumatic heart disease

##### Auscultation procedure

All study participants underwent a clinical examination by auscultation. Auscultation was done in a quiet room or outside in an enclosed screen with participants having bear-chest and rested in a 45^0^ inclined examination bed. Privacy was ensured. Cardiac auscultation was done by two trained final year medical students using standard procedures. They auscultated all the 4 auscultatory areas namely the mitral area, the tricuspid area, the aortic area and the pulmonary area. The 1st and 2nd heart sounds were firstly determined and noted if these were normal. Any additional sound was noted as a positive auscultation finding. Murmurs were then defined as systolic or diastolic and where they were best heard.

##### Echocardiogram examination procedure

**Fig. 2:**
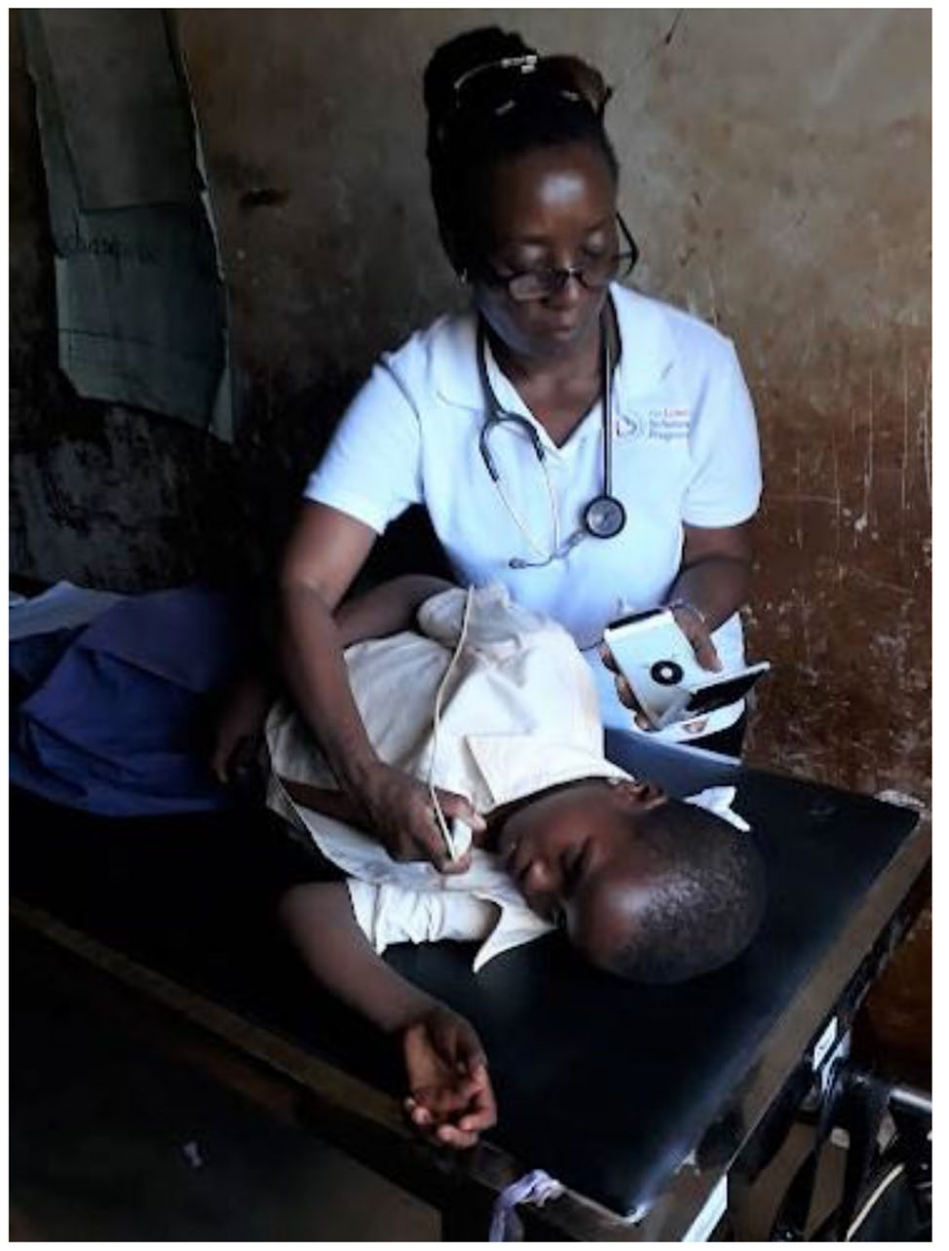
Screening using a hand-held V-Scan

After auscultation, all children underwent echocardiogram examinations using a hand-held echocardiogram machine (V-Scan, General Electric, Milwaukee WI, USA). All echocardiogram examinations were performed by two experienced Cardiologists (PC and RM) following a standard procedure, in a quiet room (Fig. 2) or outside under enclosed screen. Morphological and functional valvular lesions consistent with RHD were recorded. Morphological lesions recorded included valvular/subvalvular thickening, as well as valve deformities, including restricted valve motion, and excessive leaflet mobility. Functional lesions of any regurgitation (by color Doppler) and limited valvular opening (stenosis) were noted and recorded. At the end of each examination, the senior cardiologist determined whether the screening was “positive” or negative, and all “positive tests” were stored in the V-scan, for further scrutiny.

### Definition of a positive screening test

The modified World Heart Federation (WHF) criteria for defining RHD for individuals ≤ 20 years was used as previously described [24, 25]. Definite RHD was considered present when there was either of the following echocardiogram findings: (1) pathological mitral regurgitation and at least two morphological features of RHD of the mitral valve, (2) pathological aortic regurgitation and at least two morphological features of RHD of the aortic valve or (3) borderline disease of both the aortic valve and mitral valve. Borderline RHD was considered present when there was either of the following: (1) at least two morphological features of RHD of the mitral valve without pathological mitral regurgitation or mitral stenosis, (2) presence of pathological mitral regurgitation or (3) presence of pathological aortic regurgitation.

**Fig. 3:**
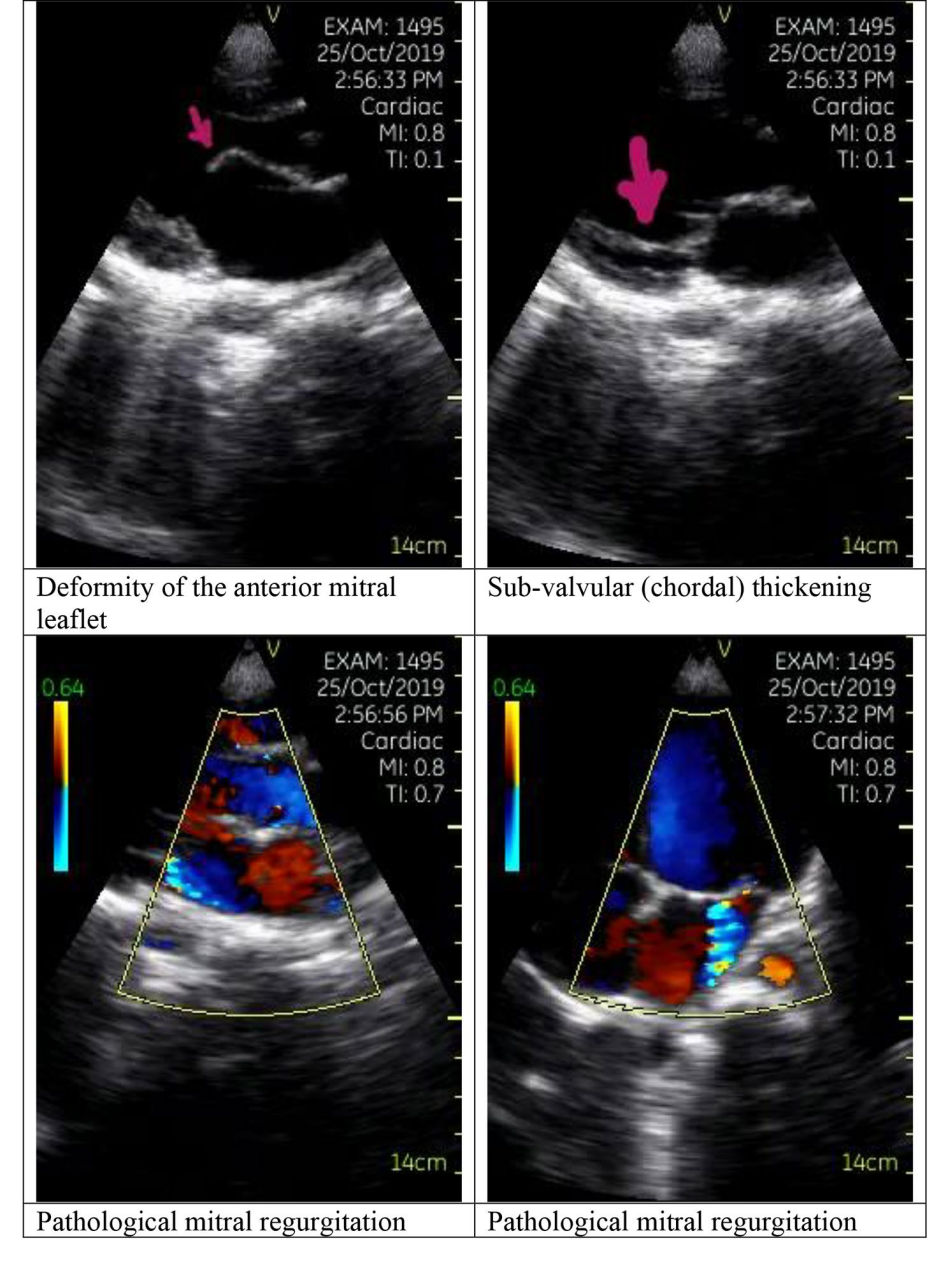
Echocardiographic findings in a child with definite RHD

**Fig. 4:**
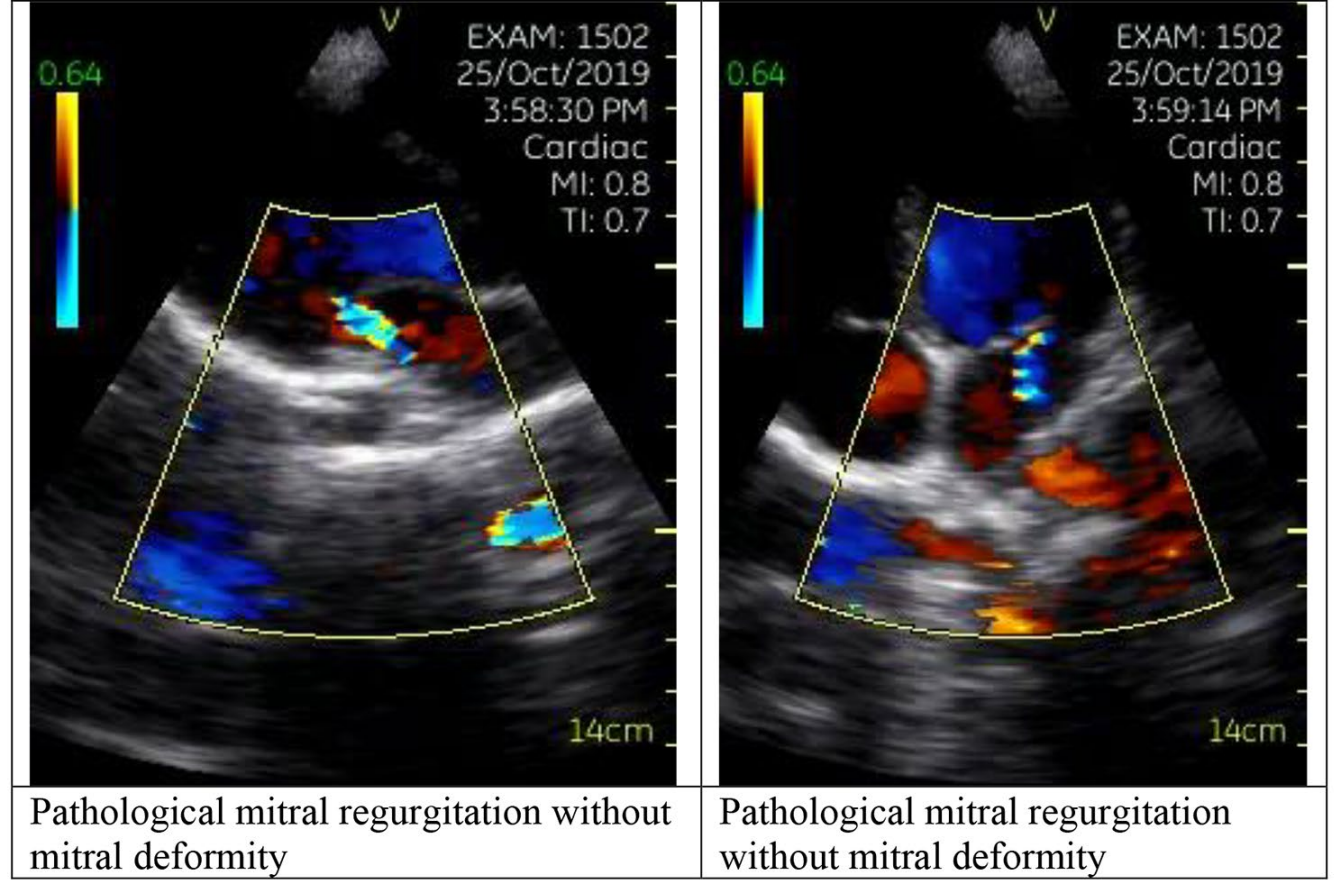
Pathological mitral regurgitation in a child with borderline RHD

Using the same criteria, pathological mitral regurgitation was defined as mitral regurgitation seen in two echocardiographic views and in at least one view, the jet length is ≥ 2 cm, while pathological aortic regurgitation was defined as aortic regurgitation seen in two views and in at least one view the jet length is ≥ 1 cm. Morphologic features include mitral valve (MV): anterior leaflet thickening, chordal thickening, restricted leaflet motion, excessive leaflet tip motion during systole; and aortic valve (AV): irregular or focal thickening, coaptation defect, restricted leaflet motion, prolapse. Examples of definite and borderline echocardiogram findings are shown in Figs. 3 and 4, respectively.

### Data management and analysis

Data entry and analysis was done using the Statistical Package for Social Sciences (SPSS) software, Version 23. All data was checked for completeness and plausibility before being entered into the data entry system. For echocardiogram findings, morphological and functional data were recorded in data collection forms on site. Then, for the second scrutiny of the initial “positive” echocardiograms, data was included in the data collection forms within 24 h after agreement as definite, borderline or normal findings between the two senior cardiologists.

Prevalence rates (with 95% confident interval, CI) of RHD were estimated in the total population and separately according to socio-demographic characteristics and according to regions, districts and the schools participated in the survey. Differences in prevalence between groups were compared using the Chi square test. Factors found to have association with RHD diagnosis in univariate analysis were entered into logistic regression model to determine independent factors associated with RHD in this study population. Then, factors known to be associated with RHD (from previous studies in the region) including maternal education, distance to the health facility, as well as number of people in the household [9, 13, 33] were included into the model to determine their influence.

### Disposal of positive screening tests

Parents/guardians of all children who participated in the study were notified of the results of their children’s screening test. Those whose children were found to have sub-clinical RHD were given further details including information about ARF prophylaxis, and were referred to the nearby health facility to continue with usual care and follow-up.

## Results

In total 6,895 children were invited to participate in the echocardiographic screening study, but only 4,738 (68.7%) were screened and 4,436 (64.3%) had complete data including the echocardiogram screening findings. Details of invited, screened and with echocardiogram examination per each school are shown in Table 2.

**Table 2:** Response rate for echocardiogram screening survey

School	Total number invited at the school	Number surveyed	Echocardiogram performed	Percent of total studied (%)
Kiromo	815	590	564	12.7
Buma	410	308	278	6.3
Kondo	340	190	178	4.0
Mloganzila	850	580	552	12.4
Masaki	526	319	274	6.2
Masanganya	330	212	188	4.2
Kaloleni	869	650	627	14.1
Mbugwe	810	545	518	11.7
Dareda Kati	750	513	458	10.3
Osteti	530	350	346	7.8
Magugu	665	481	453	10.2
**TOTAL**	**6,895**	**4,738**	**4,436**	**100%**

### Prevalence of sub-clinical RHD

Using auscultation, 53 children (out of the 4426 pupils with reported findings) were found to have a cardiac murmur, giving a prevalence of positive auscultation of 1.2% (95% CI 0.9–1.6). On screening echocardiogram, the findings showed that 367 children had trace to mild valvular regurgitation, 56 had valvular or sub-valvular thickening and 45 showed valvular deformity, Table 3. Majority of the abnormalities were detected on the mitral valve and its sub-valvular structures, while aortic abnormalities were detected in only two children (both with aortic valve thickening), and in both children the mitral valve was also involved. No child was diagnosed with congenital heart disease during the screening echocardiogram. On second analysis of the recorded V-Scan loops, 95 children were found to have sub-clinical RHD, giving a prevalence of 2.1% (95% CI 1.7–2.6). Of these, 59 were definite and the remaining 36 had borderline RHD.

**Table 3:** Findings of hand-held echocardiogram screening

Finding	Frequency*	Prevalence (95% CI)
Valvular regurgitation**	367/4433	8.3 (7.5–9.1)
Valvular and/or chordal thickening	56/4434	1.3 (1.0–1.6)
Valvular deformity	45/4433	1.0 (0.7–1.4)
Sub-clinical RHD	95/4436	2.1 (1.7–2.6)

* The denominator is not uniform as there were some few missing reports of the variables

** Any regurgitation, i.e. trace and mild regurgitation included

The prevalence of sub-clinical RHD by region and school is shown in Table 4. The highest prevalence of sub-clinical RHD was seen in Osteti primary school in Kiteto district (2.9%) and the lowest was in Dareda Kati primary school in Babati district (1.1%), both are rural schools in Manyara region. The differences in prevalence between schools were not statistically significant, p = 0.951.

**Table 4:** Prevalence of sub-clinical RHD by schools

District	School	School category	Prevalence (95% CI)
Bagamoyo	Kiromo	Semi-urban	2.3 (1.23–3.91)
	Buma	Rural	1.8 (0.59–4.15)
	Kondo	Rural	2.2 (0.62–5.65)
Kisarawe	Mloganzila	Semi-urban	2.0 (1.00–3.54)
	Masaki	Rural	2.2 (0.81–4.70)
	Masanganya	Rural	2.1 (0.58–5.36)
Babati	Kaloleni	Semi-urban	2.4 (1.35–3.93)
	Mbugwe	Rural	2.3 (1.20–4.01)
	Dareda Kati	Rural	1.1 (0.36–2.53)
Kiteto	Magugu	Semi-urban	2.2 (1.06–4.02)
	Osteti	Rural	2.9 (1.39–5.25)

### Factors associated with sub-clinical RHD

Of the socio-demographic and economic indices studied, only sex and age showed significant differences in the prevalence of sub-clinical RHD. Females were more likely to have sub-clinical RHD (2.7%) when compared to males (1.5%), and older children aged 15–16 years were more likely to have sub-clinical RHD (4.8%) than younger children aged 10–14 years (2.6%) and those aged 5–9 years (1.6%). Furthermore, children with a cardiac murmur were more likely to have sub-clinical RHD (11.3%) when compared to those without a murmur (2.0%), (Table 5). We did not find any child on long-term prophylaxis against ARF.

**Table 5:** Socio-demographic, economic and clinical characteristics of children in relation to sub-clinical RHD findings

Variable	Number in group	Number (%) with RHD in the group	p-value
**Sex**			
Males	2014	30 (1.5)	0.006
Females	2422	65 (2.7)	
**Age groups, (years)**			
5–9	1855	29 (1.6)	0.023
10–14	2429	62 (2.6)	
15–16	84	4 (4.8)	
Missing	68		
**School setting**			
Semi-urban	2196	49 (2.2)	0.683
Rural	2240	46 (2.1)	
**Living with parents**			
Yes	3733	79 (2.1)	0.732
No	689	16 (2.3)	
Missing	14		
**Head of house occupation**			
Farmer/peasant	2050	49 (2.4)	0.625
Government employed	359	5 (1.4)	
Self employed	1035	23 (2.2)	
Petty business	738	12 (1.6)	
Other occupations	206	5 (2.4)	
Missing	48		
**Mother education**			
No formal education	391	6 (1.5)	0.254
Primary education	2546	60 (2.4)	
Secondary education	878	12 (1.4)	
Above secondary	281	9 (3.2)	
Not applicable/deceased	255	6 (2.4)	
Missing	85		
**Father education**			
No formal education	258	3 (1.2)	0.414
Primary education	2390	57 (2.4)	
Secondary education	1001	17 (1.7)	
Above secondary	370	10 (2.7)	
Not applicable	325	5 (1.5)	
Missing	99		
**Head of household education**			
No formal education	326	5 (1.5)	0.580
Primary education	2468	60 (2.4)	
Secondary education	938	16 (1.7)	
Above secondary	390	8 (2.1)	
Not applicable	203	3 (1.5)	
Missing	111		
**Roofing materials**			
Iron sheets	4111	87 (2.1)	0.862
Tiles	11	0 (0.0)	
Grass	252	7 (2.8)	
Mud	51	1 (2.0)	
Missing	11		
**Wall materials**			
Bricks	3230	70 (2.2)	0.993
Mud	1011	21 (2.1)	
Wood	101	2 (2.0)	
Iron sheets	73	2 (2.7)	
Others	4	0 (0.0)	
Missing	17		
**Floor materials**			
Cement	2876	67 (2.3)	0.675
Mud	1359	25 (1.8)	
Tiles	159	3 (1.9)	
Others	19	0 (0.0)	
Missing	23		
**Number in the household**			
2–5	2088	40 (1.9)	0.375
6–10	2031	52 (2.6)	
>10	138	3 (2.2)	
Missing	179		
**Number in same room**			
1–4	4240	91 (2.1)	0.784
≥5	116	3 (2.6)	
Missing	80		
**Distance to health facility**			
Within 1 km	3006	71 (2.4)	0.527
1-5 km	1223	22 (1.8)	
>5 km	45	1 (2.2)	
Missing	162		
**Cardiac murmur**			
Yes	53	6 (11.3)	< 0.0001
No	4473	88 (2.0)	
Missing	10		

In a multivariate logistic regression analysis including sex, age groups and presence of a murmur in auscultation, all variables were found to be independently associated with the diagnosis of sub-clinical RHD, Table 6. Adding variables that were previously reported to be associated with RHD (maternal education, distance to the health facility or number of people in the household) did not alter the findings.

**Table 6:** Independent factors associated with sub-clinical RHD

Variable	Un-adjusted		Adjusted	
	OR (95% CI)	p-value	OR (95% CI)	p-value
Sex				
Males	Constant			
Females	1.82 (1.18–2.82)	0.007	1.83 (1.18–2.85)	0.007
Age groups, (years)				
5–10	Constant			
11–14	1.65 (1.06–2.57)	0.028	1.73 (1.10–2.72)	0.018
15–16	3.15 (1.08–9.18)	0.036	3.02 (1.01–9.05)	0.048
Murmur on auscultation				
No	Constant			
Yes	6.22 (2.59–14.91)	< 0.0001	5.63 (2.31–13.69)	< 0.0001

### Sensitivity of cardiac auscultation to detect sub-clinical RHD

Of the 95 children with echocardiographically diagnosed sub-clinical RHD, only 6 were also found to have a cardiac murmur, giving a sensitivity of clinical auscultation to detect sub-clinical RHD of only 6.3%. Of note, echocardiogram in this study was about 15 times more sensitive in detecting sub-clinical RHD when compared to cardiac auscultation.

## Discussion

Both the World Health Organization (WHO) and the World Heart Federation (WHF) are supporting screening for sub-clinical RHD as a critical initial step when planning for a comprehensive RHD control program in order to obtain data on the community burden of the disease [6–8]. We found the prevalence of RHD in this large representative sample of school children in Tanzania to be 2.1%. This prevalence lies within the reported RHD prevalence in SSA of 1–3% [9–18]; underlining similarities of the populations across the region.

Although the schools we studied were either semi-urban or rural located, we did not find significant different prevalence of RHD between these schools, indicating similar occurrence of the disease burden in this age group, independent of location. In fact, in a similar screening survey involving school children in Dar es Salaam (the most urbanized city in Tanzania), the prevalence of sub-clinical RHD was higher (3.4%) than the current study [14]. Moreover, our results showed no difference in prevalence between Manyara and Pwani Regions. This observation is in contrast to our anecdotal hospital data at the National Cardiac Referral Hospital (the Jakaya Kikwete Cardiac Institute) which suggests that majority of the RHD cases come from the Northern Zone (including Manyara region). Of note though, the prevalence of sub-clinical RHD was highest (2.9%) in the most remote school screened (Osteti) – a predominantly pastoralist Maasai community in Manyara region. Recently, Beaton and colleagues found that without prophylaxis, 12% of children with latent RHD in neighboring Uganda progressed to having more RHD changes within 2 years, but also 34% of these children had their RHD changes regressed to normal within the same period of follow-up [21]. It is therefore worth noting that several other factors (apart from prophylaxis) play role in determining regression as well as progression of RHD. Further research including genetic studies are therefore needed to determine these other factors which may add to our understanding of the mechanisms of progression or regression of sub-clinical RHD, and therefore add more insight to appropriate prevention strategies.

We found RHD to be more likely diagnosed in the older age children as previously reported [9, 11, 13, 14]. This is an expected occurrence as in these older children several episodes of ARF may have occurred, therefore the fibrosis, valve deformity and valve dysfunction accompanying RHD is more likely to be detected at this age [19]. Here we draw an important observation that if performed among older children and adolescents, echocardiographic screening will likely yield more positive findings, and therefore making this approach more cost effective. However, reaching out to older children and adolescents may need special efforts in a country like Tanzania, because only around 50% of adolescents proceed to secondary school education [34], therefore any school-based screening may miss half of the target population.

In this study, female children were 83% more likely to have a positive screening test for RHD when compared to male children. This finding is similar to many previous studies [9–11, 13, 14, 35]. The reasons of higher RHD among females are not well understood [35–37], although it is generally known that most of autoimmune diseases affect females more than males [38]. A recent study has however shed light on the possible reasons for female predominance in RHD, implicating Prothymosin alpha as a potential regulator of sex predisposition in RHD [39]. It has also been postulated that probably the difference seen is related to socio-cultural issues whereby males are given priority when they fall sick than females, considering the devastating household economic consequence of RHD which necessitates coping mechanisms in those at the lowest end of the socio-economic ladder [40]. The higher prevalence of RHD among girls indicate that screening for adolescent girls or young women is another strategy that may yield better cost-effective measures, taking advantage that women can be screened during pregnancy at antennal clinics. This can answer an important question on the burden of sub-clinical RHD among women, but also may serve to detect potentially significant RHD lesions, which if left unchecked can result into poor outcomes to the mother and the baby in utero [41].

In our study, the presence of a cardiac murmur was 5 times more likely to be associated with sub-clinical RHD, independent of age or sex of the child. Although this was an impressive association, the actual sensitivity of clinical auscultation to detect sub-clinical RHD in this study was very low at only 6.3%, supporting previous findings that clinical auscultation is up to 10 times less sensitive as compared to echocardiography screening [13, 25, 42]. The cardiac auscultation sensitivity was lower in our study compared to that reported by Gordon et al. in Uganda (16.4%) [25] and by Marjon et al. in Mozambique (10%) [13], likely due to the use of non-professionals (medical students) to perform auscultation in our study.

Most of the previously studied factors associated with RHD were negative in this study. Previous studies found maternal education, low socio-economic status, distance from the hospital and rural residency to be associated with RHD [9, 13, 33, 36]. We studied all these factors and none of them showed significant association with RHD. It is possible that the population of the current study was more homogenous and that these factors were somehow equally distributed in the studied population.

## Strengths and limitations

This study is the largest on RHD prevalence in Tanzania and is among the few studies that estimated the prevalence of RHD in East Africa [11, 12, 14]. Being part of an on-going program [43], this study is unique due to the fact that it will generate longitudinal data. Moreover, due to its programmatic nature involving different stakeholders, we believe this screening exercise has already raised awareness of RHD among Tanzanian government officials, healthcare workers, pupils and their parents, and teachers in the schools that were involved in the study. The on-going training component which involves health education on sore throat, ARF and RHD [43] is expected to be beneficial beyond the echocardiographic screening data alone.

We used a hand-held echocardiogram machine to detect sub-clinical RHD. The hand-held echocardiogram machine lacks the capability to perform continuous Doppler studies and therefore necessitated the use of the modified WHF to diagnose sub-clinical RHD. This may have slightly over-estimated the prevalence of sub-clinical RHD, as the hand-held echocardiogram machine is less specific and likely to include up to 13% false positives [25]. However, as previously reported by other investigators, the hand-held echocardiogram is almost as sensitive as the conventional portable echocardiogram, especially when used by experts [25–28]. Our finding of more definite RHD cases than borderline RHD cases is likely due to the fact that in this study screening and diagnoses were done by specialist Cardiologists only. Cardiologists are more likely to recognize and therefore exclude most of the false positive echocardiogram findings which could have otherwise been diagnosed as borderline disease, especially those with trace to mild mitral regurgitation (which was found in 8.3% children in this study). We therefore believe that the prevalence obtained is closely the true prevalence of sub-clinical RHD in this population.

## Conclusion

The prevalence of sub-clinical RHD among primary school children in Tanzania is 2.1%, similar to previous reports in SSA. Female sex, older age and the presence of a cardiac murmur were independently associated with the diagnosis of sub-clinical RHD in this population. Efforts to prevent and control RHD in our communities are highly warranted.

## Data Availability

The datasets generated and/or analysed during the current study are not publicly available due on-going follow-up study but are available from the corresponding author on reasonable request.

## Abbreviations

ARF: Acute Rheumatic Fever
EACoECVS: East African Centre of Excellence for Cardiovascular Sciences
RHD: Rheumatic Heart Disease
SSA: Sub Saharan Africa
WHF: World Heart Federation
WHO: World Health Organization
